# Energetics and electronic structures of perylene confined in carbon nanotubes

**DOI:** 10.1098/rsos.180359

**Published:** 2018-06-27

**Authors:** Yuya Nagasawa, Takeshi Koyama, Susumu Okada

**Affiliations:** 1 Graduate School of Pure and Applied Sciences, University of Tsukuba, 1-1-1 Tennodai, Tsukuba, Ibaraki 305-8571, Japan; 2 Department of Applied Physics, Nagoya University, Chikusa, Nagoya 464-8603, Japan

**Keywords:** carbon nanotube, perylene, encapsulation, energetics

## Abstract

The energetics and geometries of perylene encapsulated in carbon nanotubes (CNTs) have been investigated employing density functional theory using the generalized gradient approximation combined with the van der Waals correction. Our calculations show that the encapsulated perylene molecules possess two metastable molecular conformations with respect to the CNT wall, which are almost degenerate with each other. A standing conformation, with respect to the CNT wall, is the ground state conformation for a semiconducting (19,0)CNT, while a lying conformation is the ground state for a metallic (11,11)CNT. Cooperation and competition between perylene–perylene and perylene–CNT interactions cause these possible perylene conformations inside CNTs. However, the electronic structure of the CNT encapsulating the perylene molecules is found to be insensitive to the molecular conformation.

## 1. Introduction

Since the discovery of carbon nanotubes (CNTs) [1], they have been indispensable in nanoscale sciences and technologies, owing to their unique geometric and electronic properties that arise from the tubular structures of the honeycomb network of sp^2^ C atoms. CNTs are either metals or semiconductors depending on the atomic arrangement along their circumference [2,3], which makes them excellent constituent materials for next-generation semiconductor devices [4–6]. In addition to their unique electronic properties, the nanometre-scale one-dimensional spacing in CNTs allows for an interesting class of compounds composed of host CNTs and encapsulated guest materials [7–14]. In such host–guest compounds, guest materials possess different structures from those in their bulk states [7,8]. High-resolution transmission electron microscopy (HRTEM) experiments have confirmed that C_60_ [9,10] and other fullerenes [11,12] form a one-dimensional chain inside CNTs. Furthermore, because of the interaction between CNTs and fullerenes, the electronic structures of these host–guest compounds are not the simple sum of those of the constituents [15–17].

As well as fullerenes, polycyclic aromatic hydrocarbon (PAH) molecules, such as perylene, coronene, sumanene and corannulene, have also been encapsulated into single-walled CNTs. These PAH molecules also tend to possess various conformations in CNTs, which differ from those of their bulk phase. For instance, coronene, a typical aromatic molecule comprising 24 C atoms whose edges are completely terminated by H atoms [18,19], is able to form two different conformations even though it is encapsulated in a CNT of almost the same diameter: depending on the synthesis conditions, coronene possesses a stacked structure inside CNTs, where the tilt angle of the molecule is shallower than that observed in its bulk herringbone structure [20,21]; coronene can also form a double-decker structure with a lying conformation with respect to the CNT wall [22,23]. In addition, perylene exhibits two different molecular conformations, that is, stacking and lying conformations, in CNTs [24]. However, the physical and chemical mechanisms of the multiple conformations of PAHs in CNTs are still unclear.

The purpose of this work is to computationally investigate the detailed energetics of a perylene molecule encapsulated into a metallic (11,11)CNT and a semiconducting (19,0)CNT to determine the physical and chemical mechanisms of the various metastable conformations of PAH inside CNT that are experimentally observed. Our first-principles total-energy calculations reveal that cooperation between the encapsulation energy of each PAH molecule in the CNTs and the intermolecular interaction results in a potential energy landscape with a double minimum structure in terms of the molecular orientation to the CNT wall. The lying molecular conformation energetically competes with the stacking conformation with a tilt angle of 70°, which well explains the experimental results. Furthermore, the electronic structure of the CNTs encapsulating perylene weakly depends on the molecular orientation.

## 2. Material and methods

All calculations were performed within the framework of density functional theory [25,26] using simulation tool of atom technology (STATE) [27] code. To calculate the exchange–correlation energy among the interacting electrons, we used the generalized gradient approximation with the Perdew–Burke–Ernzerhof functional [28]. The weak dispersive interaction between perylene and the CNTs was treated using the vdW-DF2 functional with the C09 exchange–correlation functional [29,30]. Ultrasoft pseudopotentials generated using the Vanderbilt scheme were used to describe the electron–ion interactions [31]. The valence wave functions and deficit charge density were expanded using plane-wave basis sets with cut-off energies of 25 and 225 Ry, respectively, which gave sufficient convergence of the relative total energies of carbon-related materials [32,33]. Integration over the one-dimensional Brillouin zone was performed using two equidistant *k* points along the CNT. The atomic structures of the CNTs containing perylene were fully optimized until the force acting on each atom was less than 1.33 × 10^−3^ HR/au.

In this work, to determine whether the electronic structure of CNTs affects the molecular conformation of perylene inside a CNT, we considered metallic (11,11) and semiconducting (19,0)CNTs, both of which possessed the same diameter of 1.49 nm. Because the periodicity of CNTs is inherently incommensurate with that of perylene chains, we considered the structural model in which the formation energy *E* is decomposed into the encapsulation energy *E*_encap_ and intermolecular interactions energy *E*_inter_,

$$E=E_{\mathrm{encap}}+E_{\mathrm{inter}}.$$

**Figure 1.**
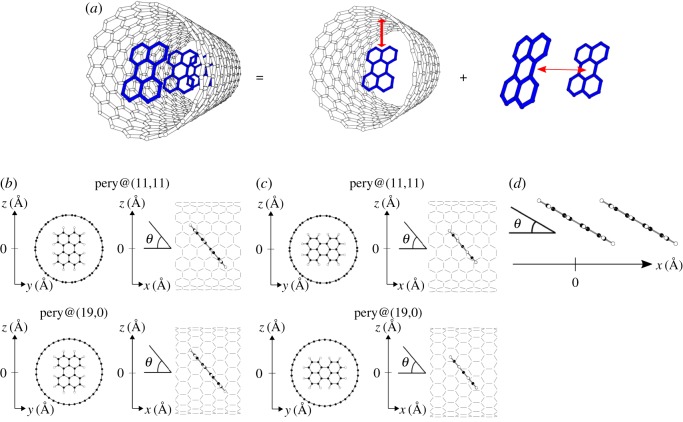
(*a*) A structural model to calculate the formation energy of a CNT encapsulating a perylene chain, which is decomposed into the encapsulation energy of perylene in the CNT and the interaction energy between adjacent perylene molecules. White and blue cylinders denote the covalent networks of the CNT and perylene. Structural models to calculate the encapsulation energy of perylene in (11,11) and (19,0)CNTs in which perylene is rotated by an angle *θ* with respect to its *C*_2_ axis (*b*) parallel and (*c*) perpendicular to the zigzag edges. (*d*) A structural model for investigating the interaction between adjacent perylene molecules. Black and white circles denote C and H atoms, respectively.

The encapsulation energy was evaluated by

$$E_{\mathrm{encap}}=E_{\mathrm{CNT}/\mathrm{perylene}}-E_{\mathrm{CNT}}-E_{\mathrm{perylene}},$$

where *E*_CNT/perylene_, *E*_CNT_ and *E*_perylene_ denote the total energies of a CNT encapsulating perylene, an isolated CNT and an isolated perylene molecule, respectively (figure 1*a*). To estimate the encapsulation energy of each perylene molecule, the perylene was encapsulated every 1.23 and 1.28 nm for (11,11) and (19,0)CNTs, respectively, which corresponded to the quintuple and triple periodicities of these CNTs. In the CNTs, the spacing *d* between CNT and perylene and the tilt angle *θ* with respect to the CNT wall were optimized (figure 1*b*,*c*). For the intermolecular interaction energy, the energy was evaluated for the equilibrium intermolecular spacing under each tilt angle (figure 1*d*).

## 3. Results and discussion

**Figure 2.**
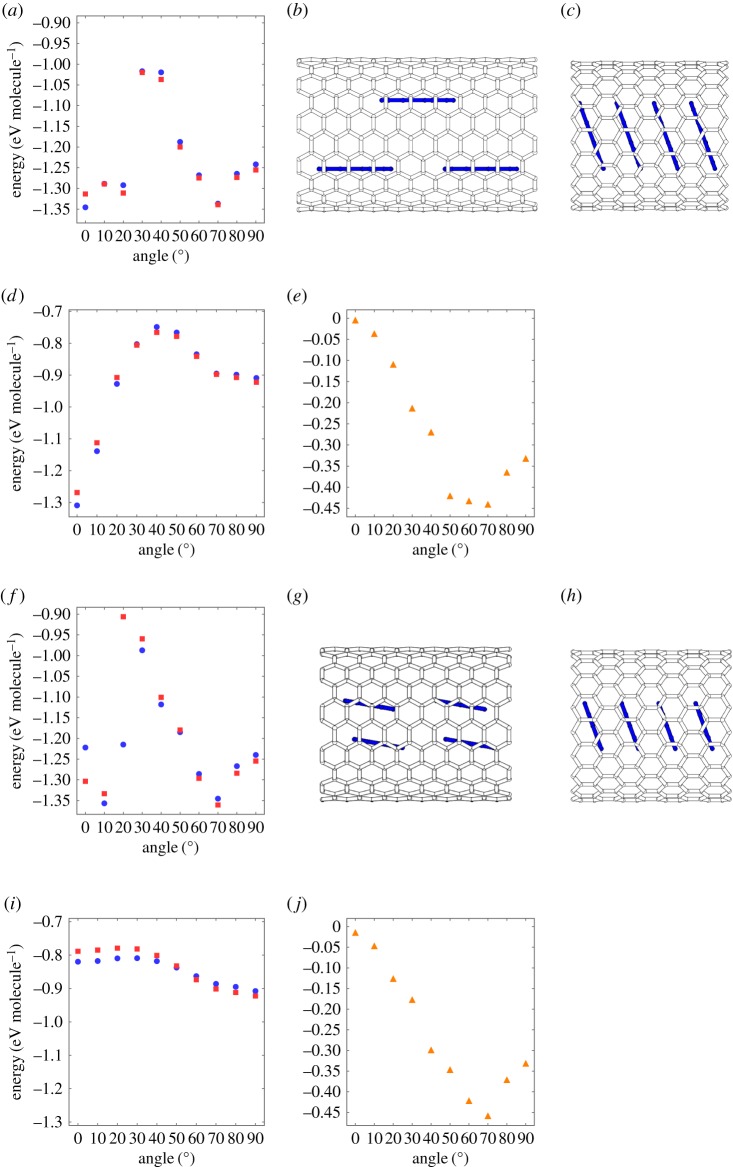
(*a*) The formation energies of (11,11) and (19,0)CNTs encapsulating perylene as a function of the rotational angle *θ* with respect to the *C*_2_ axis parallel to the zigzag edges of perylene. Optimized structures of the ground state perylene arrangements in (*b*) (11,11)CNT and (*c*) (19,0)CNT. Decomposed interaction (*d*) between CNT and perylene and (*e*) between perylene molecules as a function of the rotational angle *θ* with respect to the *C*_2_ axis parallel to the zigzag edges of perylene. (*f*) The formation energies of (11,11) and (19,0)CNTs encapsulating perylene as a function of the rotational angle *θ* with respect to the *C*_2_ axis parallel to the armchair edges of perylene. Optimized structures of the ground state perylene arrangements in (*g*) (11,11)CNT and (*h*) (19,0)CNT. Decomposed interactions (*i*) between CNT and perylene and ( *j*) between perylene as a function of the rotational angle *θ* with respect to the *C*_2_ axis parallel to the armchair edges of perylene. White and blue cylinders denote the covalent networks of CNT and perylene, respectively. Blue circles, red squares and orange triangles denote the energies associated with perylene-(11,11)CNT, perylene-(19,0)CNT and a perylene pair, respectively.

Figure 2*a* shows the formation energy of perylene chains inside the (11,11) and (19,0)CNTs as a function of the tilt angle of perylene. The encapsulated perylene was rotated with respect to the *C*_2_ axis parallel to the zigzag edges of perylene (figure 1*b*). Encapsulated perylene chains possessed two energy minima at the tilt angles of 0° and 70°: the formation energies of the two energy minima were degenerate with each other and weakly depended on the CNT species. For the (11,11)CNT, a tilt angle of 0° (a lying and double-decker arrangement; Figure 2*b*) was observed for the ground state while a tilting arrangement of 70° was the metastable state with an energy difference of 9 meV. In contrast, for the (19,0)CNT, the energy of the stacking arrangement (figure 2*c*) was 26 meV lower than that of the lying and double-decker arrangement. This fact indicated that the perylene chain can possess two energetically stable molecular conformations inside a CNT with a diameter of 1.49 nm, which is in agreement with the experimental results [24]. Regarding the encapsulation energy per length, we found that the encapsulation of the standing conformation is larger than that of the lying conformation by approximately twofold. Thus, the molecular conformation inside CNTs may depend on the number of perylene molecules or the perylene density encapsulated in a CNT: perylene relatively prefers the lying conformation under the high-density condition, while it prefers the standing conformation under the low-density condition.

To provide further insight into the energetics of the formation energy of the perylene chain inside CNTs, we decomposed the formation energy into the perylene–CNT interaction (figure 2*d*) and perylene–perylene interaction (figure 2*e*). For the perylene–CNT interaction, the lying molecular arrangement was the ground state molecular conformation with an energy of 1.3 eV for both CNTs, because of the large contact area between perylene and the CNT wall causing a strong *π*–*π* interaction. The encapsulation energy monotonically increased with an increase of the tilt angle up to 50°, and then decreased with a further increase of the tilt angle. For angles of 70° or larger, the energy exhibited a shallow local minimum where the energy was 0.4 eV higher than that with a tilt angle of 0°, because of the CH—*π* interaction [34–36]. However, the perylene–perylene interaction exhibited an energy minimum at the tilt angle of 70° where the adjacent perylene molecules were arranged in AB stacking, as in the case of graphite, which caused the largest *π*–*π* interaction between the perylene molecules. Thus, the two metastable molecular conformations in CNTs were ascribed to the delicate balance among *π*–*π* interactions between perylene molecules and between perylene and the CNT, and the CH–*π* interaction between the perylene edges and the CNT.

As for the molecular rotation with respect to the *C*_2_ axis parallel to the armchair edges (figure 1*c*), the perylene also exhibited two metastable molecular conformations in CNTs (figure 2*f* ). In contrast to the tilting along the armchair direction, a tilt angle of 10° (a lying and double-decker arrangement; Figure 2*g*) was the ground state for the (11,11)CNT, while a tilting arrangement of 70° was metastable with an energy difference of 11 meV. For the (19,0)CNT, the energy of the stacking arrangement (figure 2*h*) was 27 meV lower than the tilting conformation, of which the angle was approximately 10°. By investigating the perylene–CNT interaction in Figure 2*i*, we observed that the lying ground state conformation was less stable by 0.1 eV than the tilting conformation, because of the large spacing between perylene and the CNT wall in this molecular orientation. However, the perylene–perylene interaction exhibited a similar nature to that for the tilting along the armchair direction (figure 2*j*). These facts indicate that the molecular arrangement of encapsulated hydrocarbon molecules is determined by not only the size of the CNT and hydrocarbon molecule but also the synthesis conditions.

**Figure 3.**
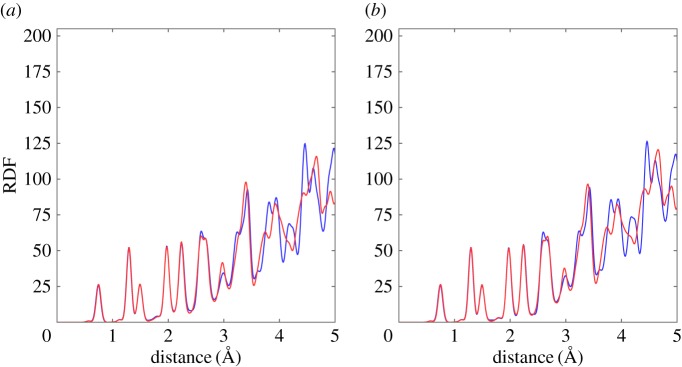
RDFs of CNTs encapsulating perylene with a tilt angle *θ* of (*a*) 0° and (*b*) 70° with respect to the *C*_2_ axis parallel to the zigzag edges of perylene. Blue and red lines denote the radial distribution functions of perylene encapsulated in (11,11) and (19,0)CNTs, respectively.

It is worth discussing why the perylene prefers the lying arrangement in (11,11)CNT but prefers the stacking arrangement in (19,0)CNT. Although both CNTs have almost the same diameter, the atomic arrangements along the circumference may be different. Figure 3 shows the radial distribution functions (RDFs) of the perylene chains encapsulated in (11,11) and (19,0)CNTs for both the lying and stacking molecular arrangements. The RDF spectra only depend on the perylene conformation and do not depend on the CNT species. Therefore, the local atomic structure of CNT was not responsible for determining the energetics of the perylene chain inside CNTs.

**Figure 4.**
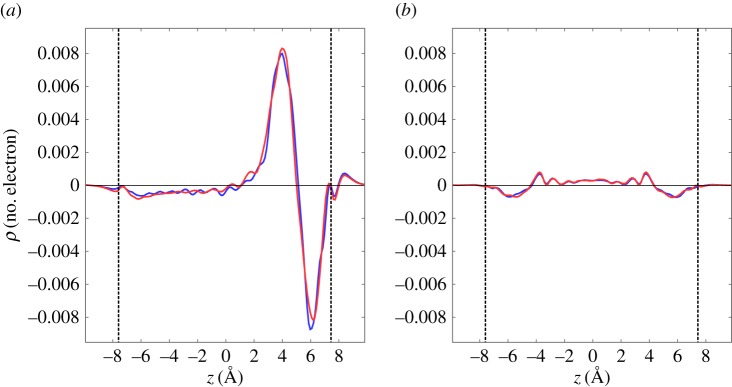
Plane averaged charge redistribution along the *z* direction inside a CNT upon perylene encapsulation with molecular tilt angles of (*a*) 0 and (*b*) 70° with respect to the *C*_2_ axis parallel to the zigzag edges of perylene. Blue and red lines denote the electron density of (11,11) and (19,0)CNTs, respectively, encapsulating perylene. The vertical dashed lines indicate the position of the CNT walls.

For a further discussion as to why the encapsulation energy in a metallic CNT is lower than that in a semiconducting CNT, we investigated the charge redistribution upon perylene encapsulation in the CNTs. Figure 4 shows the plane-averaged electron density across the CNT induced by perylene encapsulation. For the CNTs encapsulating perylene with the lying conformation (figure 4*a*), the charge density associated with the encapsulation was sensitive to the CNT, even though the RDF does not depend on the CNT species. The charge density modulation inside (19,0)CNT was slightly different from that inside (11,11)CNT. The charge density modulation was ascribed to the modulation of wave functions induced by their hybridization upon perylene encapsulation. Therefore, the charge redistribution on the encapsulation caused the small but significant energy difference of perylene encapsulation in the metallic and semiconducting CNTs with the same diameter. In contrast, for a CNT encapsulating perylene with the standing conformation, the charge redistribution upon the encapsulation was insensitive to the CNT species.

**Figure 5.**
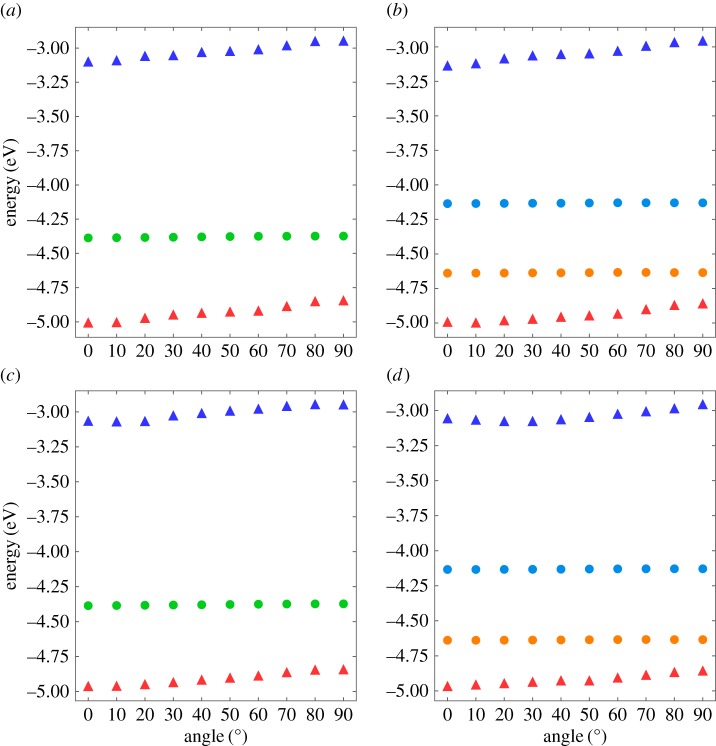
Electronic energy levels near the Fermi level of (*a*) (11,11)CNT and (*b*) (19,0)CNT encapsulating a perylene molecule as a function of the rotational angle *θ* with respect to the *C*_2_ axis parallel to the zigzag edges of perylene. Electronic energy levels near the Fermi level of (*c*) (11,11)CNT and (*d*) (19,0)CNT encapsulating a perylene molecule as a function of the rotational angle *θ* with respect to the *C*_2_ axis parallel to the armchair edges of perylene. Red triangles, blue triangles, green circles, orange circles and light blue circles denote the HO state of perylene, the LU state of perylene, the Dirac point of (11,11)CNT, the valence band top of (19,0)CNT and the conduction band bottom of (19,0)CNT, respectively. The energies are measured from that of the vacuum level.

Figure 5 shows the electronic structure of perylene encapsulated in a CNT as a function of the tilt angle. For all cases, the highest occupied (HO) and the lowest unoccupied (LU) states of perylene were located below and above the band edges of the CNT, respectively. Thus, (11,11)CNT encapsulating perylene was a metal where the carriers were distributed on the CNT, while the (19,0)CNT encapsulating perylene was a type I semiconductor where the band edges were attributed from the bonding and antibonding *π* states of the CNT. The electron states associated with the CNT were insensitive to the perylene rotation: the Dirac point of (11,11)CNT and the band edges of (19,0)CNT were retained for all tilt angles of perylene. In contrast, the HO and LU states of encapsulated perylene were slightly dependent on the tilt angle: the state for a small tilt angle was deeper than that for a large tilt angle. Additionally, the eigenvalue of the LU and HO states monotonically increased with an increase in the tilt angle. However, these states were insensitive to the host CNT species. These facts imply that the optical properties of the host CNT and guest perylene could be slightly modulated by the formation of the hybrid structures and molecular conformation.

**Figure 6.**
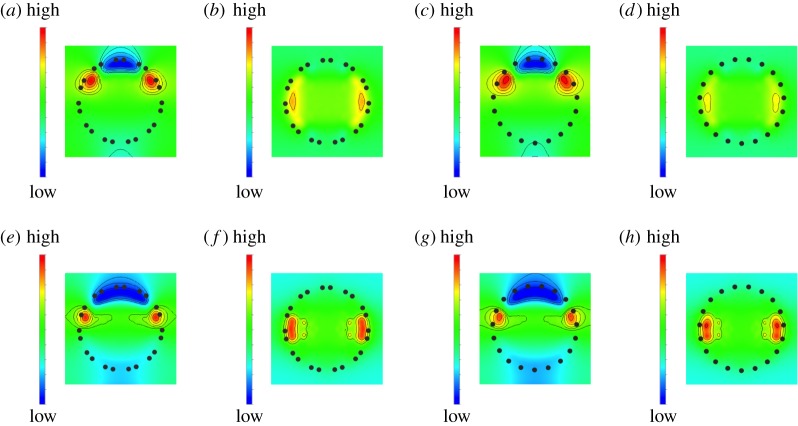
Contour plots of the electrostatic potential modulation inside (11,11)CNT upon perylene encapsulation with a rotation angle of (*a*) 0° and (*b*) 70° with respect to the zigzag edges, (19,0)CNT upon perylene encapsulation with a rotation angle of (*c*) 0° and (*d*) 70° with respect to the zigzag edges, (11,11)CNT upon perylene encapsulation with a rotation angle of (*e*) 10° and (*f*) 70° with respect to the armchair edges, and (19,0)CNT upon perylene encapsulation with a rotation angle of (*g*) 10° and (*h*) 70° with respect to the armchair edges. Blue and red regions indicate the regions where the potential energy decreases and increases, respectively, upon the inclusion.

To provide a theoretical insight into the electron states shift of perylene upon molecular rotation, we investigated the modulation of the electrostatic potential upon perylene encapsulation (figure 6): Δ*V* (***r***) = *V* (***r***) − *V*_CNT_(***r***) − *V*_pery_(***r***), where *V* (***r***), *V*_CNT_(***r***), and *V*_pery_(***r***) are the electrostatic potential of an encapsulated structure, an isolated CNT and an isolated perylene, respectively. The electrostatic potential for the lying arrangement was remarkably modulated compared with that for the standing arrangement. For the lying arrangement, the potential at the perylene shifted downward, which led to the deep LU and HO states (figure 6*a*,*c* for (11,11) and (19,0)CNTs, respectively). In contrast, for the standing arrangement, the potential modulation at the perylene was negligible (figure 6*b*,*d* for (11,11) and (19,0)CNTs, respectively).

## 4. Conclusion

We studied the energetics and electronic structures of CNTs encapsulating perylene molecules in terms of its stable molecular conformation in CNTs using first-principles total-energy calculations in the framework of density functional theory. We observed that the encapsulated perylene molecules energetically prefer both the stacked columnar arrangement, where the molecules are slightly tilted with respect to the CNT axis, and the lying and double-decker conformation, where the perylene molecule is adsorbed parallel to the inner wall of the CNT. The total energies of these two molecular conformations are almost degenerate with each other. The stacking conformation is the ground state conformation for the semiconducting (19,0)CNT, while the lying conformation is the ground state conformation for metallic (11,11)CNT. The double minimum energy profile is ascribed to the cooperation between the encapsulation energy of perylene into the CNT, which favours the lying conformation, and the intermolecular interaction energy, which favours the stacking conformation. Because the two molecular conformations and the lying and stacking conformations compete with each other, the encapsulated perylene molecules can possess both of these conformations, which may be determined by the initial condition of the encapsulation reactions. The electronic structures of the CNTs encapsulating perylene molecules appear to be a simple sum of that of each constituent with a slight modulation caused by the hybridization between the *π* states of perylene and CNTs. However, the electronic states near the Fermi level associated with the CNT and perylene are insensitive to the tilt angle of the perylene molecules.

## Data accessibility

All input data used in the manuscript are available from the Dryad Digital Repository: https://doi.org/10.5061/dryad.88h1m1h [37].
